# Physician-prescribed museum visit benefits on mental health: results of an experimental study

**DOI:** 10.3389/fmed.2025.1590145

**Published:** 2025-05-14

**Authors:** Olivier Beauchet, Adeline Moret, Melanie Deveault, Claire Thiboutot, Nicole Parent, Hélène Boyer, Kevin Galéry

**Affiliations:** ^1^Departments of Medicine and Geriatrics, University of Montreal, Montreal, QC, Canada; ^2^Research Centre of the Geriatric University Institute of Montreal, Montreal, QC, Canada; ^3^Department of Medicine, Division of Geriatric Medicine, Sir Mortimer B. Davis Jewish General Hospital and Lady Davis Institute for Medical Research, McGill University, Montreal, QC, Canada; ^4^Department of Learning and Community Engagement, Montreal Museum of Fine Arts, Montreal, QC, Canada; ^5^Médecins Francophones du Canada, Montreal, QC, Canada

**Keywords:** art, museum, experimental study, medical prescription, health

## Abstract

**Background:**

Art and cultural activities have been associated with health benefits. The effects of a physician-prescribed museum visit on the mental health of patients remain to unclear. This study aims to examine the effects of a single visit at the Montreal Museum of Fine Arts (MMFA, Montreal, Quebec, Canada), prescribed by a physician, on the wellbeing and quality of life of patients living in Montreal (Quebec, Canada).

**Methods:**

The study used a pre-post intervention, non-randomized single-arm, experimental design with prospective data collection. A total of 86 patients (mean age 51.5 ± 17.6, 82.6% female) completed the study. The intervention consisted in a single MMFA visit prescribed by a primary or secondary care physician. Well-being and quality of life were assessed before and after the MMFA visit using validated self-questionnaires completed on a web-based platform including the Warwick-Edinburgh Mental Well-being Scale (WEMWBS) and the EuroQol-5D (EQ-5D), respectively. Participants’ baseline characteristics were also recorded.

**Results:**

There was a significant improvement of both WEMWBS (≤0.001) and EQ-5D (*p* ≤ 0.002) scores between after and before the MMFA visit. The increase in WEMWBS score was positively associated with self-reported unhappiness (coefficient of regression beta (ß) = 15.15 with 95% confident interval (CI) = [4.30,25.99] and *p* = 0.007) and the presence an acute disease (ß = 10.76 with 95% CI = [3.15,18.37] and *p* = 0.006).

**Interpretation:**

A physician-prescribed visit to the MMFA was associated with improved mental health. These results suggest that museums could play a valuable role as partners in care pathways for patients in primary and secondary healthcare settings.

**Clinical trial registration:**

https://clinicaltrials.gov/study/NCT05445453, identifier NCT05445453.

## Introduction

Art and cultural activities have been associated with positive mental health outcomes, including enhanced well-being and the prevention or mitigation of mental health disorders (1). These mental health benefits are frequently reported across observational and interventional studies, regardless of whether improving health was the explicit objective (1, 2). The burden of mental illness is increasing and is a major public health issue in high-income countries like Canada (3, 4). For instance, in 2022, it has been reported that 5 million Canadians (i.e., 18% of the total population) aged 15 and older met the diagnostic criteria for mental health problems (4). Accordingly, promoting positive aspects of mental health including the well-being and quality of life is increasingly important. This target is important because promoting the well-being may prevent the occurrence of mental illnesses or limit their worsening in patients (1–3). In addition, mental health promotion, through art and cultural activities, may save health cost (5).

Among cultural activities, visiting museums may enhance well-being and quality of life (1, 4, 6). This is supported by a growing body of international literature on the health-related benefits of museum engagement. For instance, Overgaard et al. in 2015 examined museum visitors’ perceived well-being and described the mechanisms through which cultural participation contributes to mental health (7, 8). Similarly, other studies have highlighted the therapeutic potential of museums and their integration into care pathways, particularly in the UK and Europe (9–12). Despite promising evidence, most of these studies remain observational or descriptive, with limited experimental research in clinical populations. This gap underscores the relevance of the present study, which investigates the short-term mental health impact of a physician-prescribed museum visit in a real-world care setting.

Mental health problems are prevalent in primary care and represent a significant proportion of health-seeking contacts (4, 13). They are treatable but unfortunately often untreated (4, 13, 14). In 2022, one third of Canadians who met diagnostic criteria for mental health problems and who talked to a health professional reported unmet or partially met needs for mental health care services (4). Visiting museums may serve as a complementary cultural activity that supports primary health professionals in addressing patients’ mental health needs (2). However, integrating it into the care pathway of patients is a challenge (2, 5). The World Health Organization (WHO) has argued for more collaboration between arts, health and social care because of its potential benefits for care pathway (2).

To achieve this objective, we need proof of the feasibility of the collaboration between art and health providers, and evidence that this collaboration may benefit patients followed in primary and secondary care. The effects of a physician-prescribed museum visit on the mental health of patients remain unclear in Canada. We hypothesized that a physician-prescribed museum visit could improve well-being and quality of life of primary and secondary care patients living at home. With this in mind, we performed an experimental study aimed at examining the effects of a single visit at the Montreal Museum of Fine Arts (MMFA, Montreal, Quebec, Canada), prescribed by primary and secondary care physicians, on the mental health (i.e., well-being and quality of life) of patients living at home in Montreal (Quebec, Canada).

## Methods

### Design and population

The study is an experimental study using a pre-post, non-randomized, single arm design (i.e., participants served as their own control) with a prospective data collection. Primary and secondary care physicians who recruited the patients for the study were members of a Canadian physician association called “Médecins francophones du Canada.” This association is committed to rallying physicians and healthcare organizations around goals that promote quality medicine with human values to meet the needs of patients and communities. A total of 17 primary and secondary care physicians participated in the recruitment of participants.

To be included in the study, patients had to be aged 18 and over, have internet access where they lived, understand and write French or English, and sign a written consent form to participate in the study. The exclusion criteria were having dementia and concomitant participation in another experimental study. During a regular consultation, physicians informed their patients who met the selection criteria that a clinical study on the mental effects of an MMFA visit had been launched and was recruiting participants. For patients who agreed to participate, physicians completed a recruitment form that collected the patient’s contact information and the selection criteria. Following this consultation, these patients were contacted by a research team member of the AgeTeQ laboratory (localized at the Researcher center of the university geriatric institute of Montreal, Montreal, Quebec, Canada) to verify the selection criteria, present the objective and methodology of the study, answer their potential questions about the study, and obtain their written consent. If a patient did not meet the selection criteria or disagreed to take part in the study, they still benefitted from the MMFA visit.

**Figure 1:**
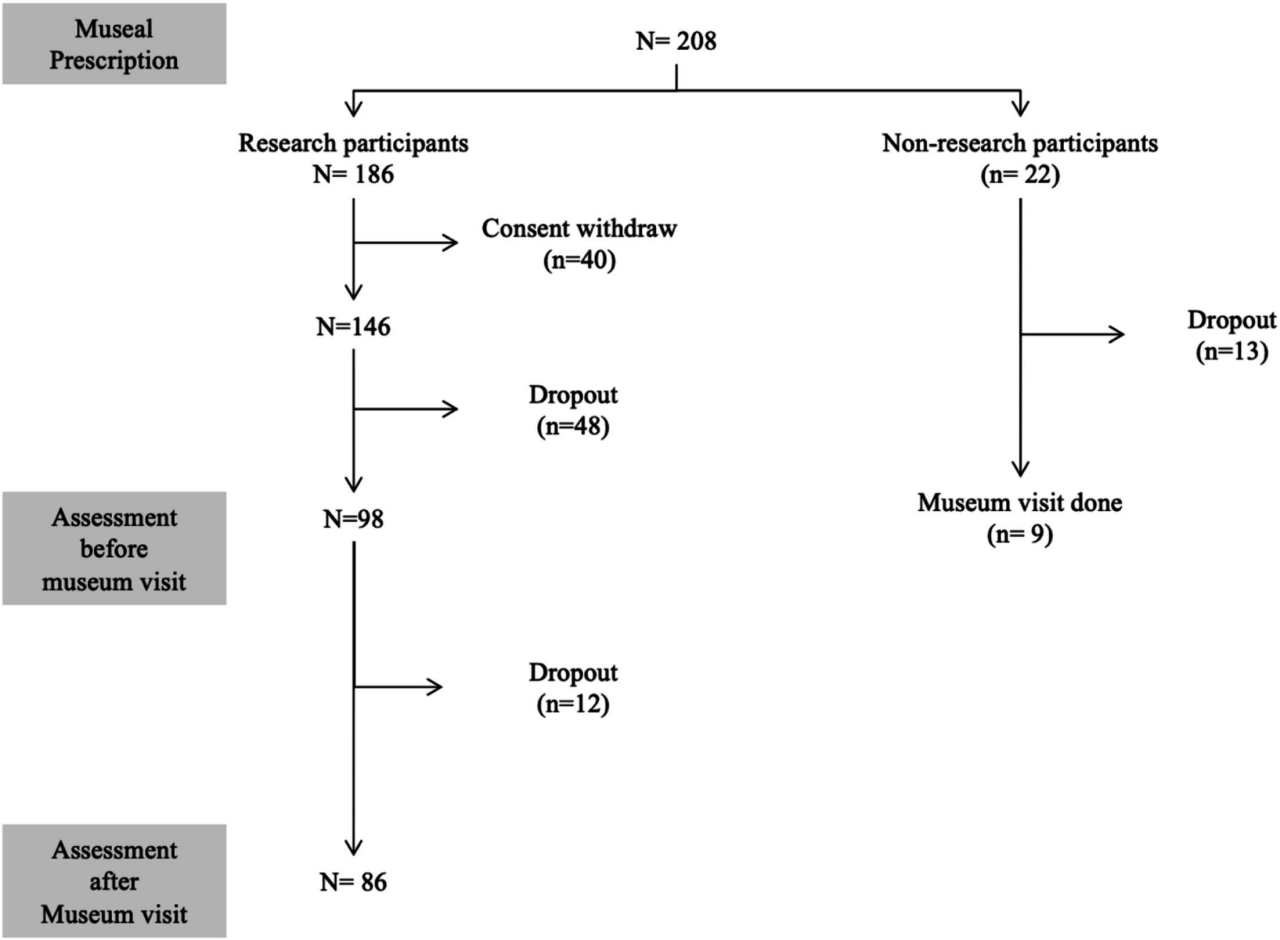
Consort flow diagram detailing selection and follow-up of participants.

On the 208 patients informed by physicians and who met the selection criteria, 186 (89.4%), agreed to participate in the study, signed a consent form and received a prescription for going to the MMFA. 22 (10.6%) patients refused to participate in the study after the phone call with the research team member of the AgeTeQ laboratory. Regardless of this refusal, the physician-prescribed MMFA visit was still valid and they were proposed to go the MMFA. Only 9 (41%) of them went to the MMFA for a visit. Among the subset of 186 research participants, 40 (21.5%) withdrew their consent before going to the MMFA. Of the 146 remaining research participants, 48 (25.8%) did not show up to the MMFA on time (i.e., within the 3 months after the physician consultation) and 98 (52.7%) participants completed their first assessment and went to the MMFA, but 12 of them (6.5%) did not complete the second assessment. Thus, 86 (46.2%) completed the full procedure of the study. Figure 1 shows the flow diagram of recruited participants.

### Assessment

Recruited patients were asked to complete a series of self-administered questionnaires within 2 days prior to the MMFA visit and within 3 days following the MMFA visit. All questionnaires were available on a secure web-based platform, and participants completed them at home online *via* smartphone, tablet or computer as self-evaluation. The time frame for questionnaire completion was deemed important to obtain the best possible representation of participants’ feelings before and after the MMFA visit, as well as to assess the impact of the museum visit. The participants were blinded to the results of all assessments. The date and time of questionnaire completion were automatically recorded through the web-based platform, allowing verification that participants respected the two-day window before and the three-day window after the museum visit. Only participants who completed both questionnaires within these specified timeframes were included in the final analysis (n = 86). Attendance at the MMFA was confirmed through a combination of methods: (1) The prescription was exchanged for museum tickets and then compile and send to the research team, allowing to track visits; and (2) cross-validation with questionnaire timestamps and, when necessary, direct follow-up with participants. This approach ensured that all included participants attended the museum and completed both assessments within the designated windows.

Before going to the MMFA, participants were first asked to complete the CESAM questionnaire (15). This questionnaire consists of 20 items exploring age; sex; nutritional status; social resources; number of medications taken daily; vision, hearing and memory concerns; mood; activities of daily living and instrumental activities of daily living; physical activity and history of previous falls. Items were presented in closed-ended format (i.e., yes or no, or requiring a specific answer). The CESAM provided two complementary summary scores: (1) A global frailty score ranging from 0 (i.e., best health and functional condition) to 18 (i.e., worst health and functional condition), and (2) categorization of health condition in terms of levels of frailty: vigorous (score 0–3), mild frailty (score 4–7), moderate frailty (score 8–12), and important frailty (>12). In addition, information about their medical condition prompting the physician consultation was collected and classified in two categories: psychiatric vs. organic disease, and acute vs. chronic disease. In our study, “psychiatric disease” refers to conditions related to mental disorders, which may overlap with certain forms of psychiatric disability. Information on whether the museum visit was alone or accompanied and if it was the patient’s first visit to a museum, was also collected.

Secondly, the well-being was assessed using the Warwick-Edinburgh Mental Well-being Scale (WEMWBS) (16). The WEMWBS covers various aspects of positive mental health (positive thoughts and feelings), with scores ranging from 14 (none of the time) to 70 (all the time).

Thirdly, quality of life was assessed using EuroQol-5D (EQ-5D) (17), which consists of two parts: a questionnaire of five questions with scores ranging from 1 (no issues) to 5 (worst issues), yielding a total score ranging from 0 (no issues) to 25 (worst issues), and a visual analog scale rating participant health from 0 (worst health imaginable) to 100 (best health imaginable). Participants completed the WEMWBS and EQ-5D scales a second time within 3 days after their MMFA visit, under the same conditions as the first assessment, at their home.

### Intervention

The intervention consisted of a free, self-guided visit to the Montreal Museum of Fine Arts (MMFA). Participants could visit the MMFA alone or with family members or friends. The museum prescription provided free admission for up to two adults and two individuals aged 20 and under, including the participant. Visits could take place on any day during opening hours and granted access to both the museum’s permanent and temporary exhibitions. Each prescription entitled the participant to a single visit per year.

### Standard protocol approvals, registrations, and patient consents

Participants were recruited only after obtaining written informed consent for the research. The local ethics committee of the recruitment center (CIUSSS Centre-Sud-de-l’Île de Montréal) approved the project.

### Statistics

The participants’ baseline characteristics were summarized using means and standard deviations (SD) or frequencies and percentages, as appropriate. Changes in scores between after and before the museum visit for well-being and quality of life were calculated from the formula: ((score after − score before) / ((score after + score before) / 2)). Comparisons were conducted using paired *t*-test. Given our sample size (*n* = 86) and the use of within-subject comparisons, this parametric approach was considered appropriate and robust to moderate violations of normality. Formal tests of normality were not performed, but visual inspection of residuals did not suggest substantial deviations. Multiple linear regressions were performed to assess the association of changes in WEMWBS and EQ-5D scores (used as dependent variables and separate models for each variable) with medical conditions (psychiatric disease and acute disease used as independent variables) adjusted for participants’ baseline characteristics. Linear regression was chosen for its ability to adjust for multiple covariates and to provide interpretable estimates of association. This method is widely accepted in pre-post study designs for analyzing continuous outcome changes. *p*-values less than 0.05 were considered as statistically significant for linear regressions. All statistical analyses were performed using SPSS (version 27.0; SPSS, Inc., Chicago, IL).

## Results

Table 1 display the baseline characteristics of participants. There was a significant improvement in the WEMWBS score (*p* ≤ 0.001) and the EQ-5D score for its questionnaire part (*p* = 0.002) and its visual analog part (≤0.001) between after and before the museum visit (Table 2). Changes in scores between after and before the museum visit for WEMWBS (6.41 ± 14.03) were not significantly different compared to those of the EQ-5D questionnaire score (7.98 ± 19.4 with *p* = 0.483) and the EQ-5D visual analog score (10.12 ± 30.56 with *p* = 0.246). There was also no significant difference of the changes in scores between the EQ-5D questionnaire score and EQ-5D visual analog score (*p* = 0.581). The linear regressions showed that the change in the WEMWBS score between before and after the MMFA visit was associated with an unhappy status (regression coefficient ß = 15.15, 95% confident interval (CI) = [4.30;25.99], *p* = 0.007) and having an acute disease (ß = 10.76, 95% CI = [3.15;18.37], *p* = 0.006) (Table 3). There were no other significant associations.

**Table 1:** Baseline characteristics of participants (*n* = 86).

Variables	Value	[95% CI]
Age (years), mean ± SD	51.5 ± 17.6	[47.7;55.5]
Female, *n* (%)	71 (82.6)	[79.5;84.2]
Caucasian^*^, *n* (%)	51 (59.3)	[49.4;71.0]
Home support^†^, *n* (%)	4 (4.7)	[1.1;9.5]
Polypharmacy^‡^, *n* (%)	10 (11.6)	[8.8;13.2]
Walking aid, *n* (%)	2 (2.3)	[0.8;4.6]
ADL score (/6)^||^, mean ± SD	5.9 ± 0.7	[5.7;6.0]
IADL score (/4)^§^, mean ± SD	3.8 ± 0.6	[3.7;4.0]
Unhappy^¶^, *n* (%)	7 (8.1)	[2.3;14.5]
Practice of physical activity^**^, *n* (%)	73 (84.9)	[76.4;92.3]
History of falls in the past 12 months, *n* (%)	12 (14.0)	[6.7;22.2]
Frailty score^††^		
Mean ± SD	8.5 ± 6.2	[7.2;9.9]
Vigorous, *n* (%)	26 (30.2)	[20.0;40.2]
Mild frailty, *n* (%)	19 (22.1)	[12.6;30.7]
Moderate frailty, *n* (%)	2 (2.3)	[0.1;5.8]
Severe frailty, *n* (%)	39 (45.3)	[34.8;56.7]
Medical condition for museum prescription, *n* (%)		
Psychiatric disease^‡‡^	75 (87.2)	[80.0;94.1]
Acute disease^||||^	68 (79.1)	[70.3;87.8]
Went to the museum alone, *n* (%)	18 (20.9)	[12.6;30.7]
First visit to the museum, *n* (%)	19 (22.1)	[13.9;32.5]

SD, Standard deviation; ADL, Activities of daily living; IADL, Instrumental activities of daily living; ^*^Versus non-Caucasian groups; ^†^Receiving help from family, friend or professional for daily living activities; ^‡^Number of therapeutic classes taken daily ≥ 5; ^||^Ranging from 0 (dependent) to 6 (independent); ^§^Ranging from 0 (non-autonomous) to 4 (autonomous); ^¶^Based on an answer to the question “How do you feel today?” with three possible answers, including unhappy, happy, neither one nor the other; ^**^Regular physical activity (walking, bicycle, etc.) at least 1 h per week in the past month; ^††^Mean score calculated from computerized self-administered questionnaire composed of 20 questions providing a score ranging from 0 (vigorous) to 18 (severe frailty) and frailty stages are categorized as vigorous (score 0–3), mild frailty (score 4–7), moderate frailty (score 8–12) and severe frailty (score >12); ^‡‡^Regardless of the type of psychiatric disease and vs. participants with organic diseases; ^||||^Regardless of the type of acute disease and vs. participants with chronic diseases.

**Table 2:** Comparisons of mean values of well-being and quality of life scores between before and after the museum visit (*n* = 86).

Variables	Museum visit		*p*-value^*^
	Before	After	
	(*n* = 86)	(*n* = 86)	
Warwick-Edinburgh Well-being scale (/70)^†^, mean ± SD	49.0 ± 9.4	52.1 ± 9.1	**≤0.001**
EQ-5D scale, mean ± SD			
Questionnaire score (/25)^‡^	7.8 ± 2.3	7.2 ± 2.1	**0.002**
Visual analog scale (/100)^||^	68.3 ± 21.6	74.1 ± 18.1	**≤0.001**

SD, Standard deviation; EQ-5D, EuroQuol 5D; ^*^Comparison based on paired t-tests; ^†^Ranging from 14 (i.e., none of the time) to 70 (i.e., all the time); ^‡^Ranging from 0 (no problem) to 25 (unable to do); ^||^Ranging from 0 (the worst health condition) to 100 (the best health condition); significant *p*-values (i.e., <0.05) in bold.

**Table 3:** Multiple linear regressions showing the association between change in wellbeing and quality of life score between before and after the museum visit (dependent variable) and baseline characteristics of participants (independent variables) participant’s (*n* = 86).

Variables	Warwick-Edinburgh well-being scale			EQ-5D scale					
				Questionnaire score			Visual analog scale		
	β	[95% CI]	*p*-value	β	[95% CI]	*p*-value	β	[95% CI]	*p*-value
Psychiatric disease^||^	4.38	[−4.44;13.19]	0.326	−0.44	[−13.29;12.41]	0.946	11.49	[−8.34;31.31]	0.252
Acute disease^§^	10.76	[3.15;18.37]	**0.006**	10.62	[−0.48;21.72]	0.060	7.68	[−9.43;24.80]	0.374
Age	−0.05	[−0.22;0.13]	0.583	−0.24	[−0.49;0.02]	0.069	0.39	[−0.03;0.78]	0.052
Female	−4.00	[−11.68;3.69]	0.303	−5.08	[−16.28;6.12]	0.369	4.62	[−12.65;21.90]	0.596
Caucasian^*^	−3.84	[−9.90;2.23]	0.211	−0.18	[−9.02;8.66]	0.968	−13.65	[−27.28;−0.02]	0.050
Frailty score^†^	−0.28	[−0.80;0.25]	0.300	−0.08	[−0.85;0.69]	0.837	−0.52	[−1.70;0.66]	0.384
Unhappy^‡^	15.15	[4.30;25.99]	**0.007**	15.13	[−0.68;30.95]	0.060	−8.17	[−32.56;16.22]	0.507
Museum visit alone	−4.05	[−11.26;3.16]	0.266	6.24	[−4.27;16.75]	0.241	−5.60	[−21.82;10.61]	0.493
First museum visit	1.37	[−5.48;8.22]	0.692	0,11	[−9.88;10.10]	0.982	−7.57	[−22.98;7.83]	0.330

β, Coefficient of regression beta; CI, Confidence interval; ^*^Versus non-Caucasian groups; ^†^Mean score calculated from computerized self-administered questionnaire composed of 20 questions providing a score ranging from 0 (vigorous) to 18 (severe frailty); ^‡^Based on an answer to the question “How do you feel today?” with three possible answers, including unhappy, happy, neither one nor the other; ^||^Regardless of the type of psychiatric disease and vs. participants with organic diseases; ^§^Regardless of the type of acute disease and vs. participants with chronic diseases; significant *p*-values (i.e., <0.05) in bold.

## Discussion

This study examined the short-term impact of a physician-prescribed museum visit on patients’ well-being and quality of life. Its findings indicate that the physician-prescribed MMFA visit improved both well-being and quality of life in patients to the same extent. In addition, the improvement in well-being was greater among patients with acute diseases who were unhappy compared to those without these medical conditions. These findings align with previous studies showing that arts and cultural engagement can positively influence mental health across various populations and settings.

The improvement in well-being and quality of life following the MMFA visit is consistent with previous experimental studies (1, 2, 6–10, 18–20). It is important to note that some of the referenced studies involved participatory art activities, where participants actively engaged in creating art. In contrast, our study focused on a free exploration of art during a museum visit. While both forms of engagement have demonstrated mental health benefits, they may involve different underlying mechanisms, such as expression and social interaction in participatory activities vs. esthetic appreciation and contemplation in receptive ones. Clarifying this distinction helps position our study within the broader spectrum of museum-based interventions. A recent literature review confirmed that museums improved visitor’s wellbeing (21). However, this review did not provide information about the timeframe for these benefits to appear. A unique aspect of our study is demonstrating that the enhancement of well-being was observed after a single museum visit. Few studies have examined the timing of the effect of museum art-based activities (2, 18), with one study specifically focusing on this issue (18). It was an experimental study involving older community dwellers, which showed that enhanced well-being during a 3-month cycle of art-based activities at a museum was observed early in the cycle, particularly during the first workshop (18). In contrast, quality of life improved gradually and became significant only after 2 months of museum activities, with the significant change observed only with EQ-5D questionnaire. This temporal discrepancy in benefits was attributed to the fact that well-being and quality of life are two related concepts with distinct differences. Well-being tends to focus more on the individual’s internal state of happiness and contentment, while quality of life encompasses a broader range of factors influencing overall satisfaction and fulfillment in life. However, in our study, both well-being and quality of life improved simultaneously immediately after a single MMFA visit. This finding was assessed using the EQ-5D, a widely validated, generic instrument designed to capture quality of life across diverse populations and health conditions (17). Its use does not require disease-specific classification, making it particularly suited for heterogeneous clinical samples such as ours.

The difference between these two studies may be attributed, firstly, to the populations studied. In our study, participants were patients of primary or secondary care physicians with a mean age around 52, whereas in the previous study, they were older community dwellers with a mean age 72. Secondly, the museum activity differed. We examined a single museum visit rather than a 3-month cycle of weekly art-based activity carried out at the museum. Thirdly, in our study, the museum activity was prescribed by a physician with the specific aim of health benefit, while in the previous study, the museum activity was presented as a leisure activity without health benefits in mind. Fourthly, in our study the MMFA was prescribed by a physician, potentially suggestion a placebo effect driven by psychological factors such as patient expectations, beliefs, and perceptions about the treatment. These psychological factors can influence the patient’s subjective experience of disease, leading to a perceived improvement.

Understanding how museum activities, such as visits, may improve wellbeing and quality of life is complex and not fully understood (2). Our findings are consistent with a broader international literature showing the therapeutic potential of museums, not only as sources of esthetic experience, but also as structured spaces for psychosocial care and well-being promotion (9–11). These works, alongside national initiatives such as the “Museums for Health and Wellbeing” alliance in the UK (12), support the integration of museums into public health strategies. It has been suggested that a variety of activities carried in museums may have a calming, restorative effect that helps visitors regain cognitive and emotional effectiveness (21). Another explanation considers the museum as providing has an esthetic experience (22). This experience involves a subjective encounter with beauty or art that stimulates one’s senses, emotions, and intellect in a profound and meaningful way (22, 23). It involves perceiving and appreciating the qualities, forms, or expressions of objects, ideas, or experiences that evoke a sense of pleasure or fascination.

Our study underscores that the enhancement of well-being was greater among patients with acute diseases and unhappiness compared to their counterparts who did not face these health problems. Several explanations can be proposed. First, individuals experiencing acute illness and unhappiness may have a significantly lower baseline level of well-being compared to those who have not faced such health problems. Therefore, any improvement, no matter how small, can be perceived as more significant and impactful. Second, going through periods of acute illness or unhappiness can lead individuals to develop a deeper appreciation for their health and overall well-being. As they recover or find ways to cope with their situation, they may develop a newfound gratitude for life and a deeper understanding of the importance of mental and physical health. Third, overcoming adversity can enhance resilience and coping skills. Patients who have faced acute illness and unhappiness may develop stronger coping mechanisms and a greater sense of inner strength, which can contribute to an overall sense of well-being. Fourth, experiencing acute illness or unhappiness can prompt individuals to reevaluate their priorities and perspective on life. They may gain a deeper understanding of what truly matters to them and focus more on activities and relationships that bring them joy and fulfillment, leading to an overall enhancement of wellbeing.

Some limitations need to be discussed regarding our study. Firstly, the study employed a pre-post, non-randomized single-arm experimental design, in which participants served as their own controls. While randomized controlled trials (RCTs) are often considered the conventional standard for evaluating intervention effects at the population level, they are not the only valid design. Alternative approaches, such as within-subject designs or non-concurrent multiple baselines, can offer strong experimental control and are particularly appropriate when investigating personalized or context-dependent interventions, as in arts and health research. Our chosen design allowed us to capture individual change over time in a real-world setting and remains well aligned with the study’s exploratory and feasibility-oriented objectives. Secondly, the study population skewed heavily toward females, comprising over 80% of participants. The benefits of engaging with art are not inherently tied to gender. However, individuals, regardless of gender, may experience art differently based on factors such as cultural background and life experiences. From a young age, individuals are often socialized differently based on their sex and gender. Cultural expectations, norms, and stereotypes regarding appropriate behavior, roles, and responsibilities may vary between males and females. This socialization can shape their attitudes, beliefs, and behaviors as they navigate various aspects of life. Thirdly, while the mean age of participants was relatively young (51.5 years), a significant proportion presented moderate to severe frailty. This observation may appear counterintuitive but reflects a broader clinical reality as frailty is not exclusive to older adults and can also affect younger individuals living with chronic diseases, psychiatric conditions, or psychosocial vulnerabilities. Fourthly, the heterogeneity of medical conditions among participants is another limitation. We used a simplified classification system (psychiatric vs. organic; acute vs. chronic) to enable statistical analysis, but we acknowledge that this approach may not fully capture the complexity of participants’ health profiles. Definitively, future studies should consider larger and more stratified samples to allow for more nuanced subgroup analyses. Fifthly, the study was designed to capture the immediate effects of a single museum visit, with assessments conducted within a few days before and after the intervention. While this approach allowed us to observe short-term changes in well-being and quality of life, it does not inform us about the persistence or long-term impact of these effects. Future studies should consider longer follow-up periods and repeated interventions to better understand the duration and potential cumulative benefits of museum-based activities. Sixthly, a high number of withdrawals occurred at various stages of the study, and while the reasons were not systematically collected, they likely reflect logistical challenges, variable motivation, or changes in health status. In addition, since museum visits were offered independently of study participation, some patients may have chosen to opt out of the research component. This introduces a potential self-selection bias, as participants who completed the full procedure may have been more motivated or predisposed to benefit from the intervention. These factors may limit the generalizability of the findings. Seventhly, potential biases such as the Hawthorne effect and test–retest bias must also be considered. Participants may have altered their responses due to the awareness of being part of a study (Hawthorne effect), despite completing the assessments independently and anonymously at home. In addition, the short time interval between pre- and post-assessments may have introduced a test–retest bias, although both the WEMWBS and EQ-5D scales are designed to capture current mental states rather than long-term traits. These biases may have influenced the magnitude of the observed effects and should be explored further in future research. In addition, we did not conduct post-hoc analyses, as our study was based on *a priori* hypotheses and pre-specified outcomes. Introducing additional exploratory analyses without a clear theoretical framework may increase the risk of spurious associations and undermine statistical validity. While post-hoc analyses may be useful in larger or exploratory studies, we believe they were not warranted in the present context. Future research with larger samples may allow for more detailed subgroup or post-hoc explorations. Furthermore, assumptions of normality were not formally tested, as both the paired t-test and linear regression are robust to moderate deviations from normality, particularly in samples larger than 30. Given our sample size (n = 86), and the use of within-subject comparisons, the application of parametric tests was considered appropriate. Visual inspection of residuals did not reveal substantial deviations from normality.

In conclusion, this study provides preliminary evidence that a physician-prescribed museum visit can improve short-term mental health outcomes. These findings highlight the potential for cultural institutions like museums to play a meaningful role in public health and primary care. Confirming our findings with stronger study designs like RCT and examining the long-term effects of museum visit, as well as explore their integration into broader healthcare strategies are essential step forward.

## Data Availability

Data will be made available on request sent by email to OB (olivier.beauchet@umontreal.ca).
